# New Synthetic Cathinones and Phenylethylamine Derivatives Analysis in Hair: A Review

**DOI:** 10.3390/molecules26206143

**Published:** 2021-10-12

**Authors:** Vittorio Bolcato, Claudia Carelli, Alessandra Radogna, Francesca Freni, Matteo Moretti, Luca Morini

**Affiliations:** Legal Medicine and Forensic Sciences Unit, Department of Public Health, Experimental and Forensic Science, University of Pavia, Via C. Forlanini 12, 27100 Pavia, Italy; bolcatovittorio@yahoo.it (V.B.); claudia.carelli01@gmail.com (C.C.); rad.ale90@gmail.com (A.R.); frafre93@gmail.com (F.F.); matteo.moretti19@gmail.com (M.M.)

**Keywords:** NPS, synthetic cathinones, phenylethylamine and derivatives, hair analysis

## Abstract

The analysis of psychoactive substances in hair is of great importance for both clinical and forensic toxicologists since it allows one to evaluate past and continuative exposure to xenobiotics. In particular, a new challenge is represented by new psychoactive substances: Among this new class of drugs of abuse, synthetic cathinone and phenethylamine derivatives are often detected in biological samples. Hence, there is a growing need to develop new analytical procedures or improve old ones in order to conduct evaluations of these emerging substances. This study is a systematic review of all the instrumental and experimental data available in the literature. A total of 32 articles were included in the review. Acidic solvents proved to be the most reliable solutions for extraction. Gas chromatography and liquid chromatography coupled to tandem mass spectrometric and high-resolution mass spectrometric systems represent the majority of the involved instrumental techniques. Sensitivity must be maintained at the pg/mg level to detect any occurrences up to occasional consumption. In total, 23 out of 32 articles reported real positive samples. The most frequently detected substance in hair was mephedrone, followed by butylone, methylone, MDPV, and α-pyrrolidinophenone-type substances.

## 1. Introduction

New psychoactive substances (NPS) are a growing concern worldwide today. Over 830 new substances are currently being monitored by the European Monitoring Centre for Drugs and Drug Addiction (EMCDDA) [1]. The chemical diversity of synthesized NPS reflects entrepreneurial mechanisms and attempts to circumvent legislative control [2]. Currently, among this huge pool of substances (synthetic cannabinoids, synthetic cathinones, ketamine-derivatives, phenethylamines, piperazines, new designer benzodiazepines, plant-based substances, and other ring-substituted substances), cathinone derivatives, synthetic cannabinoids, and synthetic opioids are among the most commonly encountered species [3]: The first ones, in particular, represent the major class of NPS on the recreational drug market [4], with relevant considerations to be made because they are also known to potentially be much more potent than their analogues [5].

Since NPS findings are growing every day, the need for sensitive, reliable, and reproducible techniques to detect and identify these substances in a variety of different matrices is frequently encountered, both for clinical and post-mortem toxicology purposes [3].

Hair testing has been successfully utilized for monitoring past and/or continuative use of classical substances of abuse and it is used to monitor drug usage during rehabilitation programs, in post-mortem cases, in workplace drug testing, in driving license regranting, and in child custody cases [6]. Moreover, hair has been proposed and is currently under evaluation as a potential alternative/complementary matrix in doping control tests [6].

The wide diagnostic time-window covered by the keratin matrix allows retrospective investigation of drug prevalence and diffusion. Furthermore, a quali-quantitative analysis could provide important information about the frequency of use, even among NPS [7]. This fact, together with the increased sensitivity and specificity achieved by the latest-generation liquid chromatography and gas chromatography systems coupled with tandem mass spectrometry and high-resolution instruments (LC–MS/MS and GC-MS/MS), allows scientists to improve the existing methodologies and increase the number of monitored substances by including NPS [7,8]. 

Though the accumulation rate of such substances in hair is not yet known, concentrations of parent drugs in this matrix are generally higher than those measured for their metabolites, in contrast to other tissues and biological fluids of forensic interest, such as urine. Hence, hair analysis represents an important alternative to the most common samples, whenever the substance to be detected is unknown.

In this study, we report a review of the existing literature on new synthetic cathinone and phenylethylamine analysis in the keratin matrix, by focusing on the different preanalytical and instrumental approaches, as well as on the different substances detected and quantitated in hair.

## 2. Results and Discussion

### 2.1. Monitored Substances

Among 32 articles (Table S1 from Supplementary Materials), 12 studies have developed methods for the detection of both classical drugs of abuse and new psychoactive substances in hair; only 7 out 12 were applied to real cases, confirming the reliability of the method. In particular, Lagoutte-Renosi et al. (2021), Rust et al. (2012), Larabi et al. (2019), and Salomone et al. (2017) found a wide number and variety of NPS in hair, confirming the strength of the developed method [7,9,10,11]. All the cited methods have been applied through an LC-MS/MS system, using different incubation and extraction methods. Larabi et al. proposed an LC-HRMS method for the detection of different classes of NPS, such as synthetic cathinones, synthetic opioids, and synthetic cannabinoids. On the contrary, Wang et al., in 2020, studied only 5-Methoxy-*N*,*N*-diisopropyltryptamine 5-MeO-DIPT in a population of 5610 drug users and found 151 positive cases (2.7%) [12]. Six studies have monitored only NPS, including Boumba et al. who included 132 NPS with an LC-MS/MS screening method [3]. 

### 2.2. Preparation and Extraction Method (Homogenization, Incubation, Extraction)

The developed extraction procedures generally included methanol or ethanol incubation for 3 to 16 h and/or sonication.

Acidified methanol appeared to be a reliable extraction solvent for phenethylamine and cathinones; in fact, Boumba et al. observed an increased extraction efficiency using 0.1 HCl methanol in comparison to pure methanol [3]; however, a two-step extraction (methanol followed by acidified methanol incubation) procedure led to good recoveries of both phenethylamines and synthetic cathinones [6,13]. Furthermore, acidic aqueous solutions provided good results in terms of better extraction efficiency and a higher sensitivity of the method, especially in the case of synthetic cathinones analyses [14]. Though liquid/liquid extraction (LLE) procedures are commonly used for hair analyses, significant matrix effects have been observed [13]. Hence, in general, acidic solvents are preserved from compound degradations.

The influence of incubation time was tested by Boumba et al. at 3 h and overnight, by maintaining a temperature at 40 °C. Since the results did not significantly differ, there is no need for a long incubation time. A sonication-assisted procedure may even shorten the extraction procedure [3]. 

Pressurized liquid extraction (PLE) coupled to a solid-phase extraction (SPE) clean-up was proposed by Montesano et al. as a sample treatment procedure for multi-class analysis of NPS in hair [13]. In comparison to classical hair digestion (i.e., NaOH), PLE seemed more appropriate for multi-class analysis considering that several compounds may not be stable under alkaline conditions (i.e., cathinones). PLE is based on extraction through solvents at a relatively high temperature and pressure. It provides several advantages over competing techniques (i.e., microwave-assisted extraction, supercritical fluid extraction) such as being time-saving, the reduced organic solvent, automation, and efficiency [13].

### 2.3. Analytical Technique

A prevalence of LC-MS/MS was observed, though GC-MS/MS has been frequently adopted for the detection of mephedrone [15,16,17]. LC-MS/MS is gradually replacing gas chromatographic techniques in both screening and confirmation procedures and is increasingly acknowledged as the technique of choice for hair analysis [18]. The LC-MS/MS method presented is a suitable procedure for the separation, detection, and quantification of synthetic cathinones and piperazines in authentic hair samples [5,19]. The separation of cathinones and amphetamine-type stimulants (ATS) was enhanced when starting the chromatographic run with 100% phase A. Furthermore, an extraction from the hair matrix with an acidic aqueous solution produced a very clean extract with a negligible matrix effect.

### 2.4. Application to Real Samples

Four out of thirty-two selected studies (13%) were based on screening procedures, without an application on real samples [3,10,13,20]; only five quali-quantitative methods (16%) did not detect any of the monitored substances in real samples [21,22,23,24,25].

Some studies carried out analyses on hair collected from other body areas: Frison et al. and Namera et al. evaluated pubic hair [26,27]; Wang et al. measured NPS in pubic and as well as axillary hair [12]; and Salomone et al. detected NPS in hair collected on different body areas [7,28]. 

An evaluation of the potential influence due to hair pigmentation was measured on rats [29,30]. In vivo studies highlighted differences between the accumulation of phenethylamines in pigmented and non-pigmented rat hair, due to the interaction with melanin. Concentrations above the cut-off have been detected only in pigmented hair, confirming the role of melanin in retaining phenethylamines in the keratin matrix. This hypothesis was eventually confirmed in human hair by Namera et al. The authors measured two synthetic cathinones, namely α-pyrrolidinobutiophenone (α-PBP) and α-pyrrolidinovalerophenone (α-PVP), in hair samples collected from drug users. The two substances have been detected in colored hair, while non-pigmented samples provided negative results, supporting the fact that pigmentation and melanin represent key factors for synthetic cathinone incorporation in hair [27].

The most-frequently detected cathinones in hair discussed in the cited manuscripts are listed in Table 1.

Overall, the most frequently detected was mephedrone. It was found in 83 cases among 12 different studies [31,32]. The concentrations were measured within a wide range (0.005–313.2 ng/mg). However, by excluding the highest concentration (313.2 ng/mg), Martin et al., in 67 cases, set the calculated average concentration at 2.9 ng/mg [16]. The high concentration of mephedrone detected could be related to chronic abuse of mephedrone [16]. 

Hence, to perform reliable quantitative analysis, limits of quantitation should be set at concentrations at least lower than 1 pg/mg. In fact, the method developed by Shah et al., which achieved an LOQ of 5 pg/mg for mephedrone in hair, allowed for the measurement of only one out of five positive cases [33]. 

The use of synthetic cathinones, especially mephedrone, among MDMA and other drugs users was confirmed by different studies [17,34,35]. Kintz et al. observed that 37% of ecstasy users also tested positive for mephedrone [36], while Larabi and co-authors detected this synthetic cathinone in 29% of drug users [11]. All these studies highlighted the importance of including synthetic cathinones hair analyses in the routine workflow of forensic toxicology labs.

Another important analytical issue is represented by the need for isomer separation. For example, 3-methylmethcathninone (3-MMC) is an isomer of mephedrone (4-MMC) [37]. An LC-MS/MS method, based on reverse-phase chromatography, usually cannot separate the two isomers. Frison et al. proposed to switch to GC-MS or, as an alternative, to extend the method to the detection of main metabolites [26]. Four different metabolites of 3-MMC, namely 3-methylnorephedrine, 3-methylpseudonorephedrine, 3-methylephedrine, and 3-methylpseudoephedrine, have been detected in pubic hair, together with the parent drug, through liquid chromatography coupled with a high-resolution mass spectrometric system (LC-HRMS) [26]. This study highlighted the importance of the latest-generation high-resolution mass spectrometric instruments in detecting potential NPS consumption through the identification of metabolites in hair. Yet, reaching high method sensitivity represents the key point whenever data on literature are scarce or unavailable. 

Several NPS were detected by Salomone et al. in 26 out of 80 (32.5%) monitored participants at different events at nightclubs and dance festivals in the New York City area [7]. Butylone, methylone, and methoxyethamine were detected, exclusively or concurrently, in 25 out of the 26 positive cases. The measured concentrations were generally high (range: 0.007–4.900 ng/mg; mean: 0.440 ng/mg; median: 0.021 ng/mg). Interestingly, among NPS detected, butylone was the only one measured at a concentration higher than 0.100 ng/mg in 9 out of 26 cases [7]. The authors did not state whether the method was able to separate butylone from ethylone, another synthetic cathinone that shares the same mass/charge ratio and MRM transitions with the latter. Hence, it is not possible to clarify whether all the positive cases should be referred to exclusively as butylone.

4-methcathinone (4-MEC) was identified in hair by seven different studies. In particular, Alvarez et al. referred to the high concentrations measured in hair (≥30.0 ng/mg) as chronic consumption rather than occasional use [38]. Pichini and co-authors were able to diagnose fetal exposure to 4-MEC after identification of this synthetic cathinone in different hair segments collected from the mother after delivery. The concentration measured in the segments ranged from 3.9 to 4.3 ng/mg [39]. 

3,4-methylenedioxypyrovalerone (MDPV) is another synthetic cathinone often studied and detected in the biological matrix, including hair. Concentrations of about 1.0 ng/mg or higher were considered as consistent with regular consumption in the study by Wyman et al. [40]. In a fatal intoxication of MDPV reported by the authors, the substance was measured in the hair at a concentration of 11.66 ng/mg; this concentration was supposed to be related to chronic exposure to synthetic cathinone. However, the study of Namera et al. observed concentrations of MDPV in hair fairly higher (ranging 300.0–350.0 ng/mg) than the one measured in the fatal intoxication. Hence, these two studies, apparently contradictory, proved that data on synthetic cathinones in hair are still too scarce to be adequately interpreted. Further, for α-PVP, different concentrations were measured in two different studies. Namera et al. reported concentrations of α-PVP ranging from 300.0 to 350.0 ng/mg, while Salomone et al. measured the same substance in hair at a concentration of about 1.0 ng/mg [27,28]. 

Lagoutte-Renosi and co-authors detected α-pyrrolidinohexiophenone (α-PHP) together with two main metabolites; in particular, a product of two hydroxylations and oxidation at the pyrrolidine ring (diOH α-PHP, <10 pg/mg), a glucuronided monohydroxylated alpha-PHP, a dihydro-alpha-PHP (reduction of the keto moiety, H2 alpha-PHP, <20 pg/mg), and its glucurinated form [9].

In accordance with the 5-MD metabolic pathway previously proposed by Michely et al. using HL-MS and in vivo studies in rats, five metabolites of 5-MD were identified in hair: A *N*-dealkyl 5-MD, two monohydroxylated 5-MD (hydroxylation occurred on aryl and alkyl), an *N*-dealkyl and hydroxy (*N*-dealkyl-HO-5MD), and *O*-demethyl 5-MD [9,41]. It was not found in its glucuronide form (*O*-demethylglucuronide 5-MD). Among 1-(1,2-diphenylethyl)-piperidine (DIP) metabolites, only hydroxylated DIP was found in hair, while tests were negative for dihydroxylated DIP and glucuronided hydroxylated DIP [9]. 

To the best of our knowledge, only Boumba et al. (for 2C-C-N-BOMe) and Palamar et al. (for 2C-B-N-BOMe) reported the detection, albeit only qualitative, of a member of the N-BOMe family in human hair, a finding that was reported only in rats in the studies of Nisbet et al. (for 2C-C) in the range of 11–143 × 10^−3^ ng/mg, and of Nieddu et al. (for 2C-B, 2C-T-2, 2C-T-7) [3,29,30,42].

### 2.5. Influence of Cosmetic Treatments and Interindividual Factors

To date, the evaluation of the potential influence of cosmetic treatments and interindividual factors, such as hair color, age, gender, BMI, etc., have not yet been studied for this class of substances. Normally, compounds with high melanin affinity, such as cocaine, are accumulated at a higher rate in black hair in comparison with white hair. The presence of melanin was seen to play a role in the link with phenethylamines [27]; however, these data must be confirmed for a greater number of substances and on a larger sample of subjects. Yet, strong cosmetic treatments may significantly impact the stability of exogenous substances in hair. For examples, polar compounds such as ethyl glucuronide, an ethanol phase II metabolite, are almost completely eliminated from the keratin matrix after bleaching [43,44]. Other treatments may hinder the detection of other compounds, such as cocaine [45]. Hence, future studies should also focus on these issues, to clarify whether synthetic cathinones and the accumulation of phenylethylamine derivatives in hair can be affected by certain factors.

## 3. Materials and Methods

All existing clinical trials published in English, enrolled through updated electronic databases (PubMed), and published up to March 2021 were examined according to the “Preferred Reporting Items for Systematic Reviews and Meta-analyses” (PRISMA) guidelines [46]. The research was performed using the (combination of) search terms “synthetic cathinones”, “mephedrone”, “phenylethylamine”, “phenylethylamine derivatives”, “NBOMe”, “LC-MS/MS”, “GC-MS/MS”, “LC-HRMS”, “GC-HRMS”, “hair”.

Literature monitoring was performed at all stages, from the initial drafting of the paper to the submission of the revised and final version. Exclusion criteria were articles not written in English, review articles, letters, and editorials. Case reports and case series were included. 

### Protocol

**Figure 1:**
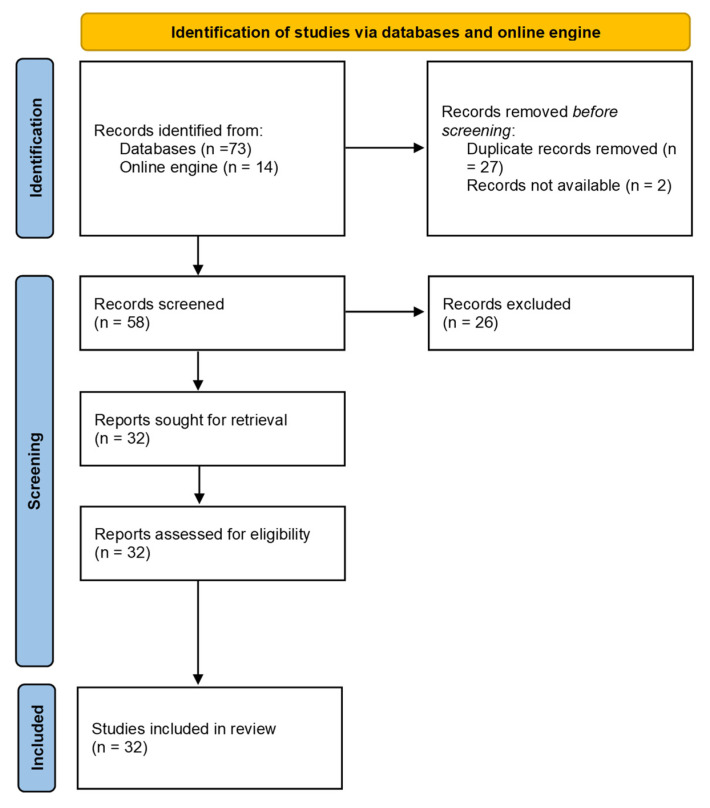
PRISMA flowchart showing the study selection process.

The electronic database search yielded 73 manuscripts, while 14 articles were added by the authors from reference lists, for a total of 87. The articles decreased to 60 after removing duplicates. Two articles were not able to be read by the authors, while 26 articles were removed because they were not considered adequate; the full texts of the remaining 32 articles were obtained. All articles were included in the systematic review (Figure 1).

For all different studies, the following parameters and results were studied: Monitored substances and detected substances, sample homogenization (cut or pulverized), sample incubation and extraction (liquid/liquid extraction, solid phase extraction, or direct injection), analytical instruments (LC-MS/MS, GC-MS/MS, LC-HRMS, or GC-HRMS), method sensitivity (LOD and LOQ), and, when available, the range of concentrations of the detected substances and their metabolites (Table S1 from Supplementary Materials).

## 4. Conclusions

To date, data concerning the detection and quantitation of synthetic cathinones and phenetylamines in hair are still scarce. Among synthetic cathinones, mephedrone is the most-frequently monitored and detected substance in hair. Other frequently identified compounds in hair are butylone, methylone, MDPV, and α-pyrrolidinophenone-type substances. Apparently, the latest-generation GC-MS/MS and LC-MS/MS instruments provide adequate sensitivity for monitoring this class of new synthetic psychoactive substances in hair. The high number of potential isomers not only requires a high-sensitivity method, but also adequate chromatographic conditions. Yet, high-resolution mass spectrometric systems could provide useful information about the presence of metabolites in the keratin matrix besides parent drugs. LODs and LOQs should be set at levels lower than 1.0 pg/mg. Moreover, data concerning the potential effect of different inter-individual factors and cosmetic treatments on the accumulation rate and stability of such substances should be extensively evaluated in future studies. Finally, the continuous and rapid changes of new psychoactive substances available on the illicit market force clinical and forensic toxicology labs to keep analytical procedures updated and to include the hair analysis in the routine workflow.

## Figures and Tables

**Table 1:** Most-frequently detected new synthetic cathinones in hair.

Substance	Number of Cases (n. of Articles)	Concentration Range in ng/mg
mephedrone	83 (12)	0.005–313.2
butylone	42 (4)	0.001–4.9
4-MEC	31 (7)	0.001–97.3
MDMC	26 (6)	0.006–21.7
MDPV	19 (9)	0.001–11.66 (−300–350 *)
α-PVP	19 (11)	0.001–1.04 (−300–350 *)
4-FA	16 (5)	0.029–7.8
MXE	15 (6)	0.03–2.93
EPH	4 (3)	0.11–1.17
cathinone	3 (3)	0.1–1.27
TFMPP	3 (3)	0.003–0.03
α-PHP	3 (3)	0.019–4.7

Legend: 4-MEC: 4-Methylethcathinone; MDMC: Methylone; MDPV: 3,4-Methylenedioxypyrovalerone; α-PVP: α-pyrrolidinopentiophenone; 4-FA: 4-Fluoroamphetamine; MXE: Methoxetamine; EPH: Ethylphenidate; TFMPP: Trifluoromethylphenylpiperazine; α-PHP: α-pyrrolidinohexiophenone. mCPP: Meta-chlorophenylpiperazine; DXM: Dextromethorphane; 2-CE: 2,5-dimethoxy-4. * Concentration not clearly reported in the article. See Table S1 from Supplementary Materials for details.

## Data Availability

The data presented in this study are available in Supplementary Material.

## Supplementary Materials

The following are available online, Table S1: Eligible studies with results included and discussed.

## Sample Availability

Samples of the compounds are not available from the authors.
