# Supplementary material for: New Synthetic Cathinones and Phenylethylamine Derivatives Analysis in Hair: A Review
Source: Molecules. 2021 Oct 12;26(20):6143. doi: 10.3390/molecules26206143 (PMC8538434; doi:10.3390/molecules26206143)
Supplement: Supplementary file 1 [file molecules-26-06143-s001.zip › molecules-1396613-supplementary.pdf]

molecules

MDPI

Table S1. Eligible studies with results included and discussed.

Authors, year [ref]	Amount of hair (mg)	Number of real cases	Real cases	Researched substances	Homogenization	Incubation	Extraction	Analytical technique	Detected molecules (concentration, ng/mg)	LOD ng/mg	LOQ ng/mg
Montesano et al., 2017 (10)	20	-	-	14 NPS (synthetic cathinones; synthetic cannabinoids) method validation	cut	PLE	SPE	LC-HRMS	2-FMC (<LOD); 4-FPP (1.05); 2-MeOMC (<LOD); Mephedrone (0.24); MXE (0.83); α-PVP (0.48); MDPV (0.75)	0.5-7 x 10^{-3} See table 2	8-50 x 10^{-3} See table 2
Gerace at al., 2014 (11)	100	1	HoA	mephedrone	cut	15 h in MeOH at 55°C	DI	GC-MS/MS	Mephedrone (0.25)	-	0.2
Martin et al., 2012 (12)	50	67	HoA	mephedrone	cut	Overnight at 40°C in Soerensen buffer	LL	GC-MS/MS	Mephedrone (n=13, 0.2-313.2; mean 26.8)	0.08	0.2
Kintz et al., 2016 (13)	-	116	112 HoA + 4 HoA, FC	mephedrone	cut	Overnight at 40°C in phosphate buffer	LL	GC-MS/MS	Mephedrone (n=28, 0.1-86.81)	20 x 10^{-3}	–
Jamey et al., 2016 (36)	20	1	HoA	3-MMC	cut	Overnight at 40°C in	LL	LC-MS/MS&	3-MMC (206.7)	0.1	0.2

						Soerensen buffer		UPLC-MS/MS			
Shah et al., 2012 (32)	50	154	volonteers	mephedrone and its two metabolites 4-methylephedrine and 4-methylnorephedrine	cut	10 min with 1 M sodium hydroxide solution	LL	LC-MS/MS	Mephedrone (n=5, in only 1, 21.11 x 10^{-3})	2.5-5 x10^{-3}	5-10 x 10^{-3}
Salomone et al., 2016 (7)	25	77	FC	26 NPS (synthetic cathinones)	cut	15 h at 55°C in MeOH	DI	UHPLC–MS/MS	4-MEC (n=1, 300 x10^{-3}); Mephedrone (n=2, 50-59 x10^{-3}); MXE (n=3, 7.7-28 x10^{-3}); α-PVP (n=1, 1040 x10^{-3}); MDMC (n=2, <LOQ-28 x10^{-3}); 4-FA (n=1, 55 x10^{-3}); MDPV (n=1, 120 x10^{-3}); DIP (n=1, 4400 x10^{-3})	0.9-17 x10^{-3}	1.8-35 x10^{-3}
Niebel et al., 2019 (4)	50	40	HoA, FC	35 NPS (synthetic cathinones)	cut	-	DI filtered	LC-MS/MS	4-BMC (n=1, 2.73); Pentedrone (n=1, 7.34); MDPV (n=3, 0.02-0.80); benzedrone (n=3, <LOQ-0.15); mephedrone (n=5, <LOQ-3.50); α-PPP (n=1, < LOQ); cathinone (n=4, 0.10-1.27); metamfepramone (n=2, <LOQ-0.01); α-PVP (n=1, < LOQ); MOPPP (n=1, 0.01)	6–52 x 10^{-3} See table 2	8-95 x 10^{-3} See table 2
Freni et al., 2019 (34)	20	17	HoA, PMC	16 synthetic cathinones	cut	16h in HCL 0.1 M at 45°C	SPE	LC-MS/MS	3,4-DMMC (n=1, 572-2800 x 10^{-3}); 4-FMC (n=1, 41.1-45.6 x 10^{-3}), 4-MEC (n=1, 591-2200 x 10^{-3}); α-PHP (n=1, 3600-4700 x10^{-3}), α-PVP (n=1, 24.4-52.8 x10^{-3}); MC (n=1, 695.6-1600 x 10^{-3}),	0.1-2.5 x 10^{-3}	1-5 x 10^{-3}

									methedrone (n=1, 1500-6200 x 10^{-3}); pentedrone (n=1, 198.4-586.2 x 10^{-3}); ethcathinone (n=1, 11 x 10^{-3})		
Alvarez et al., 2017 (37)	20	1	HoA	MDPV, 4-MEC (plus DOA and NPS)	pulverized	10 min in phosphate buffer pH 5 at 95°C	LL	LC-MS/MS	MDPV (1); 4-MEC (30); mephedrone (0.1)	0.5 x 10^{-3}	1 x 10^{-3}
Larabi et al., 2019 (10)	20	480	H (HoA and AI)	DOA and 83 NPS (synthetic opioids; synthetic cathinones; synthetic cannabinoids)	pulverized	10 min in phosphate buffer pH 5 at 95°C	LL	LC-MS/MS	mephedrone (n=24, 0.005–169); 4-MEC (n=24, 0.001–97.3); *α*-PVP (n=3, 0.001-0.14) MDMC (n=15, 0.008–21.7); MDPV (n=7, 0.001–1.5); TFMPP (n=2, 0.003-0.03); butylone (n=3, 0.001-0.17); cathine (n=3, 0.012-1.57); EPH (n=2, 0.11-0.485); m-CPP (n=1, 0.07); cathinone (n=1, 0.13); NEP (n=2, 0.015-0.64); pentedrone (n=2, 0.018-0.65), 2-CE (n=1, 0.07-0.25); mexedrone (n=1, 0.03-0.05), MC (n=3, 0.011-0.485), 6-APDB (n=2, 0.05-0.18); 3,4-MD-*α*-PHP (n=1, qualitative analysis); DXM (n=27, 0.001-8.37); MPA (n=2, 0.19-0.91); MXE (n=2, 0.13-2.93); DIP (n=1, 0.02-0.12); 4-FA (n=1, 7.5-7.8) See figure 3	0.5-2 x 10^{-3}	1-5 x 10^{-3}

Vignali et al., 2019 (31)	-	1	PMC	Synthetic cathinones	pulverized	overnight in HCL 0.1 M at 37°C	SPE	LC-MS/MS	α-PHP (1078 x 10^{-3}); α-PVP	10 x 10^{-3}	0.5
Boumba et al., 2017 (2)	20	23	PMC (HoA and suspect of abuse)	132 NPS (synthetic opioids; synthetic cathinones; synthetic cannabinoids) (only qualitative)	cut	3 h at 40°C in methanolic HCL	DI	LC-MS/MS	PMMA (n=2); cathinone (n=1); MDPPP (n=1); MeO-PCP (n=1); MPA (n=1); MDMC (n=2); PPMA (n=1); MDEC (n=3); DMA (n=3); DMT (n=3); butylone (n=1); pentedrone (n=1); α-PVP (n=2); MDPV (n=2); EPH (n=1); PV (n=1); α-PBP (n=1); PENT (n=1); 2C-C (n=1)	0,01-0,05	LOR 0,1 x 10^{-3}
Rust et al., 2012 (9)	20-30	325	HoA	DOA and NPS (synthetic cathinones) (only qualitative)	cut	–	DI	LC-MS/MS	mCPP (n=29); TFMPP (n=1); mephedrone (n=1); methylphenidate (n=16); 4-FA (n=12)	10-50 x 10^{-3}	–
Nieddu et al., 2015 (28)	100	5 (on rats)	–	11 phenylethylamine (tested on rats)	cut	24 h at 45°C in 1% methanolic HCL	DI	LC-MS/MS	PMA (0.87); PMMA (<LOQ); TMA (0.72); DMA (1.30); DOM (0.70); DOET (0.90); DOB (0.85); 2C-B (0.37); 2C-I (0.45); 2C-T-2 (0.25); 2C-T-7 (0.30)	0.03-0.07 see table II	0.09-0.2 See table II
Wang et al., 2020 (11)	50	5610	HoA	DOA and NPS (synthetic opioids; synthetic cathinones; synthetic cannabinoids)	cut	–	DI	LC-MS/MS	5-MeO-DIPT (n=151)	0,05-0,5 See table II	0.0005-0.05 See table I
Nisbet et al., 2017 (29)	40	55 (on rats)	–	25B-NBOMe; 25C-NBOMe; 25I-NBOMe	cut	12 h at 40°C in phosphate buffer pH 7.4	SPE	LC-MS/MS	2C-C (n=3, 11–143 x 10^{-3}); 25I-NBOMe (n=3, m 14–92 x 10^{-3}); 2C-B (n=3, 22-92 x 10^{-3})	3-5 x 10^{-3}	6,25-12,5 x 10^{-3}

Imbert et al., 2014 (22)	50	2	FC	DOA and NPS (17 phenethylamine)	cut	18 h at 45°C in phosphate buffer pH 5	SPE	LC-MS/MS & LC–ESI-MS/MS	-	0.005-0.030	0.05
Salomone et al., 2017 (6)	20	80	High risk population (nights)	82 substances (DOA and 26 NPS (synthetic cathinones; synthetic cannabinoids))	cut	15 h at 55°C in MeOH	DI	UHPLC–MS/MS	butylone (n=25, <7–4900 x 10^{-3}, mean 440 x 10^{-3}); MDMC (n=5; <6–98 x 10^{-3}); MXE (n=4, 3–19 x 10^{-3}); 5/6-APB (n=1; 82 x 10^{-3}); α-PVP (n=1; 6 x 10^{-3}); 4-FA (n=1; 29 x 10^{-3})	0.9-17 x10^{-3}	1.8-35 x10^{-3}
Lendoiro et al., 2017 (23)	30	16	FC	DOA, NPS (synthetic cathinones) and medicine	cut	1 h at 60° in 0,1% methanolic HCL	DI	LC-MS/MS	-	0.2-5 x10^{-3}	2-20 x10^{-3}
Kim et al., 2007 (24)	20	141	Suspected HoA	DOA and NPS (synthetic cathinones)	cut	1h at 50°C in 0.25 M methanolic HCL	DI	GC-MS/MS	-	0.002-0.024	0.01-0.08
Wyman et al., 2013 (39)	5	1	HoA	DOA, NPS (synthetic cathinones) and medicine	pulverized	1 h at 60°C in 0,1% methanolic HCL	SPE	LC-MS/MS	MDPV (11660 x10^{-3}); MDMC (1332 x10^{-3})	2 x10^{-3}	2 x 10^{-3}
Strano-Rossi et al., 2014 (13)	30	50	FC	NPS (synthetic cathinones; synthetic cannabinoids)	cut	Overnight at 45°C under sonication in MeOH or HCOOH 0.1%	DI	LC-MS/MS & UHPLC-MS/MS	MDPV (n=1, 50 x10^{-3}); 4-MEC (n=2, <LOQ-26 x10^{-3}); cathine (n=2, 100-120 x 10^{-3})	2-20 x 10^{-3}	500 x 10^{-3}
Elian et al., 2014 (20)	10	1	HoA	DOA and NPS (synthetic cathinones)	-	30 min with 0.1M sodium	SPE	LC-MS/MS	-	0,05	0,1

						hydroxide solution					
Pichini et al., 2014 (38)	-	1	FC	DOA and 4-MEC	-	1 h at 100°C in M3 buffer reagent	DI	LC-MS/MS	4-MEC (3.9-4.3)	-	0,05-0,1
Barroso et al., 2010 (19)	20	-	FC	TFMPP; mCPP; MeOPP	cut	40 min with 1M sodium hydroxide solution	SPE	GC-MS/MS	TFMPP; mCPP; MeOPP	-	50 x 10^{-3}
Vincenti et al., 2019 (21)	100	9	HoA	60 DOA and NPS (synthetic cathinones; synthetic cannabinoids)	cut	PLE	PLE	UHPLC–MS/MS	-	0.1-5 x10^{-3}	0.2-50 x10^{-3}
Frison et al., 2016 (25)	10	1	HoA	5 NPS (synthetic cathinones)	pulverized	12 h at 45° in methanol: trifluoroacetic acid 9:1	DI	Orbitrap LC-HRMS	3-MMC (25.8)	0.02	0.1
Lagoutte-Renosi et al., 2021 (8)	20	2	AI	DOA, NPS (synthetic cathinones; synthetic cannabinoids) and medicine	cut	1 h in methanol at 60°	DI	LC-MS/MS & LC-HMRS	5-MD (n=1, 6.15-12.2); DIP (n=1,27-121 x10^{-3}); EPH (n=1, 211-1170 x10^{-3}); MPA (n=1, 28-4600 x10^{-3}); 4-FA (n=1, 46-5600 x10^{-3}); 4-MEC (n=1, 36-774 x10^{-3}); MXE (n=1, 174-1010 x10^{-3}); α-PVP (n=2, 21-756 x10^{-3}); α-PHP (n=1, 19-100 x10^{-3}) and its metabolite; NEB (n=1, 176-780 x10^{-3}); mephedrone (n=1, 11-100 x10^{-3}); DMT (n=1, 9-100 x10^{-3}); MDMC(n=1, 100 x10^{-3})	5-10 x10^{-3}	-

Namera et al., 2013 (26)	0.1	4	HoA	a-PBP; MDPBP; a-PVP; MDPV; pyrovalerone	cut	20 min at 70° in NaOH 1M	SPE	LC-ESI-MS	α-PBP (n=2, 3.1-<50); α-PVP (n=4, >0.4-<350*); MDPV (n=2, >0.5-<350*)	0.2	0.5
Mannocchi et al., 2020 (30)	25	10	HoA	32 DOA and 87 NPS (synthetic opioids; synthetic cathinones; synthetic cannabinoids)	cut	1 h at 100° in M3®reagent	DI	UHPLC-MS/MS	6-APB (n=1, 0.007); mephedrone (n=1, 0.24); 5-MeO-AMT (n=1, 0.007)	0,5-6 $x10^{-3}$	1-30 $x10^{-3}$
Palamar et al., 2017 (41)	20	90	HoA	NPS (synthetic cathonines)	cut	15 h at 55°C in MeOH	DI	UHPLC-MS/MS	Butylone (n=13); MDEC (n=10); PENT (n=9); Methylone (n=3); α-PVP (n=2); 2C-B (n=1); PMMA (n=1); 4-FA (n=5); 5/6-APB (n =2); MXE (n=4); PCP (n=2); Diphenidine (n=1)	0.9-17 $x10^{-3}$	-

Legend: DOA: drugs of abuse; NPS: new psychoactive substance; HoA: history of abuse; FC: forensic cases; PMC: post-mortal cases; AI acute intoxication; H: hospitalized. Substances acronyms: 3-MMC: 3-Methylmethcathinone; MDPV: 3,4-Methylenedioxypyrovalerone; 4-MEC: 4-Methylethcathinone; α-PHP: α-pyrrolidinohexiophenone; α-PBP: α-pyrrolidinobutiophenone; α-PVP: α-pyrrolidinopentiophenone; MXE: Methoxetamine; 5/6-APB: 5/6-(2-aminopropyl)benzofuran); 4-FA: 4-Fluoroamphetamine; 5-MeO-DIPT: 5-methoxy-N,N-diisopropyltryptamine; 2-FMC: 2-fluoromethcathinone; 2-MeOMC: 2-Methoxymethcathinone; 4-FPP: 4-fluorophenylpiperazine; 2-FMC: 2-fluoromethcathinone; MOPPP: 4'-methoxy-α-pyrrolidinopropiophenone; 4-BMC: brephedrone; 3,4-DMMC: 3,4-dimethylmeth-cathinone; NEP: N-ethylpentylone; 6-APDB: 6-(2-Aminopropyl)-2,3-dihydrobenzofuran; TFMPP: Trifluoromethylphenylpiperazine; mCPP: meta-chlorophenylpiperazine; DXM: Dextromethorphane; EPH: ethylphenidate; 2-CE: 2,5-dimethoxy-4-ethylphenethylamine; MPA: methiopropamine; PV: pyrovalerone; PENT: pentylone; MDPPP: 3,4-methylenedioxy-alpha-pyrolidinopropiophenone; PMMA: p-methoxy-methamphetamine; MeO-PCP: methoxyphenylcyclidine; MC: methcathinone or ephedrone; MPA: 2-methiopropanamine; MDMC: methylone EC: ethylcathinone; PMA: p-methoxyamphetamine; PPMA: phenylpropanmethylamine; MDEC: ethylone; DMA: dimethoxyamphetamine; DMT: dimethyltryptamine; TMA: 3,4,5-trimethoxyamphetamine; DOM: 2,5-dimethoxy-4-methylamphetamine; DOET: 2,5-dimethoxy-4-ethylamphetamine; DOB: 2,5-dimethoxy-4-bromoamphetamine; 25B or 2C-B: 2,5-dimethoxy-4-bromophenethylamine; 25I or 2C-I: 2,5-dimethoxy-4-iodophenethylamine; 2C-T-2: 2,5-dimethoxy-4-ethylthiophenethylamine; 2C-T-7: 2,5-dimethoxy-4-n-propylthiophenethylamine); 25C or 2C-C: 2,5-dimethoxy-4-chlorophenethylamine; BDB: 1-(3,4-methylenedioxyphenyl)-2-butanamine; 4-MTA: 4-methylthioamphetamine; MBDB: N-methyl-1-(1,3-benzodioxol-5-yl)-2-butanamine; MeOPP: 1-(4-methoxyphenyl)piperazine; 5-MD: 5-methoxytryptamine; DIP: 1-(1,2-diphenylethyl)-piperidine; NEB: N-ethylbuphedrone; MDPBP 3,4-methylenedioxypyrrolidinobutiophenone; 5-MeO-AMT: 5-methoxy-α-methyltryptamine.
